# Nomogram-based prediction of the risk of AVF maturation: a retrospective study

**DOI:** 10.3389/fmed.2024.1432437

**Published:** 2024-11-14

**Authors:** Bin Zhao, Hui Wang, Yuzhu Wang, Shen Zhan, Pei Yu

**Affiliations:** ^1^Beijing Haidian Hospital, Peking University, Beijing, China; ^2^Tianjin Metabolic Diseases Hospital, Tianjin Medical University, Tianjin, China

**Keywords:** hemodialysis, arteriovenous fistula, fistula maturation, risk factor prediction model, nomogram

## Abstract

**Objective:**

Early identification of autogenous arteriovenous fistula (AVF) maturation failure in patients with end-stage renal disease (ESRD) is crucial, as it enables early interventions that can enhance AVF maturation rates and reduce the duration of catheter use. This study aimed to identify factors that may predict arteriovenous fistula maturation in patients undergoing maintenance hemodialysis.

**Methods:**

This retrospective study included a cohort of 532 ESRD patients who underwent AVF creation and routine follow-up at the Beijing Haidian Hospital (Haidian Section of Pecking University Third Hospital) from August 2018 to March 2022. A total of 532 patients were randomly divided into a training cohort (389 cases) and a validation cohort (143 cases). Patients in each cohort were categorized into mature and non-mature groups based on whether they met clinical or ultrasound criteria 3 months after AVF creation. The timing of early intervention for non-maturation AVF was preliminarily discussed after a risk prediction nomogram for non-maturation in newly AVF creation was constructed.

**Results:**

Of the 532 patients, 379 (73.24%) achieved fistula maturation at 3 months postoperatively. We randomly divided the total study population using computer-generated randomization into a training cohort (*n* = 389) and a validation cohort (*n* = 143) in an approximately 7:3 ratio. Analysis of the training cohort revealed that the anastomotic diameter (anas1), the internal diameter of the anastomotic vein (V1), brachial artery blood flow (F1) at 1 month, and brachial artery blood flow (F3) at 3 months postoperatively were associated with AVF maturation. A maturation nomogram was developed for the training cohort, yielding an area under the curve (AUC) of 0.938 (95% confidence interval [CI], 0.908–0.967), with a sensitivity of 0.911 and a specificity of 0.856. The model was validated in the validation cohort, showing an AUC of 0.927 (95% CI, 0.879–0.975), with a sensitivity of 0.870 and specificity of 0.886. The calibration curve showed strong agreement between nomogram predictions and actual observations.

**Conclusion:**

The anastomotic diameter (anas1), the internal diameter of the anastomotic vein (V1), brachial artery blood flow (F1) at 1 month, and brachial artery blood flow (F3) at 3 months postoperatively can predict the unassisted maturation of AVF.

## Introduction

The incidence and prevalence of end-stage renal disease (ESRD) are increasing globally (1, 2). Clinical practice guidelines recommend autogenous arteriovenous fistula (AVF) as the preferred vascular access for hemodialysis, as it is associated with fewer complications, longer access patency, and a lower risk of mortality (3–5). However, a major drawback of AVF is the risk of maturation failure, with approximately 20 to 60% of AVFs failing to mature adequately for effective dialysis (6–8). This high rate of AVF maturation failure often results in the prolonged use of central venous catheters (CVCs), which are associated with complications such as infection, thrombosis, and central vein stenosis (4, 9).

This situation highlights the importance of early identification of ESRD patients at risk for AVF maturation failure and the timely implementation of effective interventions to promote AVF maturity. Vascular remodeling and the establishment of rapid blood flow are the key factors determining AVF maturation (10–12). Many studies have shown that significant vascular remodeling often occurs within the first 4 weeks after AVF creation (5, 13, 14). However, the utility of postoperative ultrasound measurements at 4 weeks to assess AVF immaturity and guide intervention strategies remains unclear in the existing literature. This gap indicates the need for reliable diagnostic tests and predictive models to stratify the risk of AVF maturation failure. It is widely recognized that predictive models can be used to predict outcomes in patient cohorts. Particularly, in the context of vascular access, the ability to predict AVF maturation may allow for more personalized clinical practices, enhance treatment planning, and ultimately improve the rate of maturation. Thus, this study aimed to further investigate whether the postoperative ultrasound measurement of the blood flow and fistula diameter at 4 weeks is useful in predicting the likelihood of AVF maturity. Additionally, we generated a nomogram to help clinicians to decide whether and when to perform an assisted maturation intervention.

## Methods

This retrospective, single-center cohort study was conducted at the Beijing Haidian Hospital (Haidian Section of Pecking University Third Hospital) and adhered to the ethical principles outlined in the Declaration of Helsinki. This study was also approved by the Hospital Ethics Committee before patient recruitment (BHHMEC-XM-2022-18).

### Study design and patient population

A total of 532 patients who underwent initial AVF surgery between August 2018 and March 2022 were retrospectively included. All patients received monthly supervision and follow-up for at least 3 months using color Doppler ultrasound and telephone interviews. No additional surgical interventions were performed during this period. The potential clinical and laboratory potential predictors were collected from electronic medical records (EMRs) and previous paper records at the Beijing Haidian Hospital (Haidian Section of Pecking University Third Hospital). The inclusion criteria were as follows: (i) stage 5 or 5D chronic kidney disease (CKD); (ii) fistula with a cephalic vein and a radial artery; (iii) sessions of ultrasound monitoring at least twice during the 3 months after AVF placement; (iv) no previous AVF; and (v) standardized ultrasound examinations before surgery. The minimum vessel criteria for AVF creation were an arterial diameter > 1.5 mm and a vein diameter > 2.0 mm. The exclusion criteria were as follows: (i) patients under the age of 18 years old; (ii) Patients receiving both peritoneal dialysis and hemodialysis; and (iii) AVF in the upper arm.

### Candidate predictive factors for AVF maturation

Demographics included sex, age, comorbidities (diabetes, hypertension, and vascular disease), smoking status (ever vs. never), and dialysis duration.

Laboratory examinations involved hemoglobin (Hb), albumin (ALB), serum calcium, and serum phosphorus, which were collected within 1 week before surgery.

Vascular characteristics included the type of fistula (forearm or upper arm), its location (right or left), and the position of the catheter.

Postoperative ultrasound data (1-month post-surgery) were collected at 1 month and included measurements of the brachial artery diameter (B1), brachial artery blood flow (F1), anastomotic diameter (anas1), internal diameter of anastomotic vein (V1), and internal diameter of anastomotic artery (A1).

### Standards and definitions

Two standard definitions were used to describe AVF outcomes (15, 16). A “mature AVF” was defined as having a flow rate of 500 mL/min, a main body diameter of at least 5 mm, and a depth of no more than 0.6 cm from the skin, with successful two-needle cannulation and the ability to provide dialysis at least three times a week (15). An “immature AVF” was defined as failing to meet the standards of a mature AVF after 12 weeks. The main clinical performance parameters of an immature AVF included insufficient blood flow (below 200 mL/min) and difficulty in achieving access by a nurse specialist.

Duplex ultrasound surveillance was conducted by a skilled nephrologist on the brachial artery, the radial artery, the anastomosis site, and the cephalic vein. The cephalic vein was measured 5 cm from the anastomosis, the radial artery was measured 1 cm from the anastomosis, and the brachial artery diameter and blood flow were measured 5 cm above the elbow. To prevent measurement biases, the same nephrologist, trained in vascular ultrasound, conducted all examinations, and each patient underwent three successive measurements, which were averaged.

### Statistical analysis

Categorical variables were presented as frequencies and percentages, and a chi-squared test was performed to assess significance. Continuous variables were expressed as mean ± SD, and a T-test was conducted to determine. A *p*-value of <0.05 was considered statistically significant. We used a computer-based randomization method to divide the total study population into a training cohort (consisting of 389 participants) and a validation cohort (consisting of 143 participants) in an approximate 7:3 ratio.

To identify the combination of variables that would provide the highest predictive efficiency for constructing binary logistic regression, we first conducted univariate logistic regression analysis. Variables with *p* < 0.05 in univariate regression were then included in a multivariate binary logistic regression model, and a nomogram was developed based on these significant parameters. The performance of the nomograms was evaluated using the area under the receiver operating characteristic curve (AUC) and calibration, assessed through calibration plots and the Hosmer–Lemeshow calibration test, all conducted in R (version 4.2.1). The statistical analysis involved using packages such as rms, Resource Section, PredictABEL, nricens, pROC, regplot, and survivalROC.

## Results

### Study flow and patient characteristics

**Figure 1:**
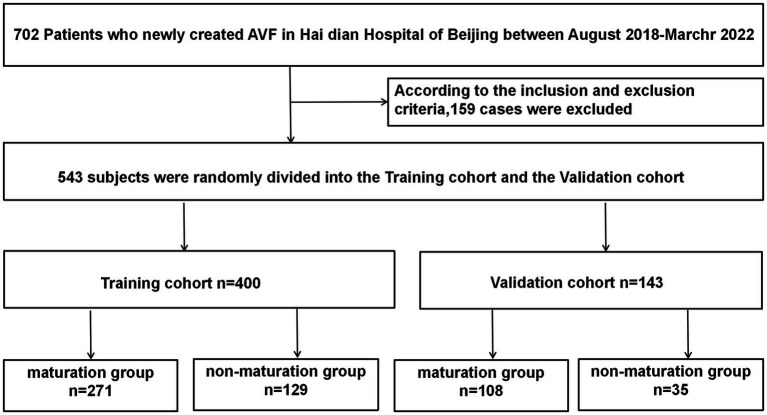
Flowchart of study participants in the training and validation cohorts.

We initially examined 702 patients who underwent AVF procedures at our institution between August 2018 and March 2022. Of these, 170 patients were excluded for the following reasons: 132 patients had AVF resections or reconstructions, 8 patients received both peritoneal dialysis and hemodialysis, 22 patients lacked follow-up postoperative information, 2 patients were cognitively impaired investigations, and 6 patients were lost to follow-up due to renal transplantation or death. Ultimately, 532 patients met the eligibility criteria and were enrolled in the study (Figure 1).

Three months post-AVF creation, patients were categorized into maturation and non-mature groups based on whether they met the clinical criteria for AVF maturity. Of the 532 patients, 389 were randomly assigned to the training cohort and the remaining 143 to the validation cohort. We first compared the baseline characteristics of the training and validation cohorts (Table 1). Compared to the validation cohort, the training cohort showed no significant differences in several factors, including age, history of antiplatelet aggregation drugs, history of CCB use, history of ACE inhibitors/angiotensin receptor blockers ACEI/ARB, smoking history, average blood pressure, and various ultrasound-measured vascular parameters including anastomotic diameter, venous diameter, arterial diameter, and brachial artery blood flow at 1 and 3 months post-surgery (*p* > 0.1). There were no significant differences between the two groups regarding red blood cells, blood creatinine, blood urea nitrogen, blood phosphorus, blood albumin, blood hemoglobin, total cholesterol, total protein, activated partial thromboplastin time (APTT), and fibrinogen levels (*p* > 0.1). However, the validation cohort had a higher proportion of individuals with diabetes (*p* < 0.05), a lower proportion of forearm fistula surgeries, and a greater proportion of male participants (*p* < 0.1).

**Table 1:** Baseline characteristics of the study cohort.

Characteristic		Training cohort *n* = 389	Validation cohort *n* = 143	*p*
Age (y)		57.019 ± 13.488	58.314 ± 13.386	0.777
Sex	Male	226 (58.10%)	95 (66.43%)	0.081
	Female	163 (41.90%)	48 (33.57%)	
Maturation group		271 (69.67%)	108 (75.52%)	0.186
Diabetes		146 (37.53%)	68 (47.55%)	0.037
Hypertension		328 (84.32%)	119 (83.22%)	0.758
Left		287 (73.78%)	103 (72.03%)	0.686
Forearm arm		337 (86.63%)	117(81.82%)	0.164
Taken antiplatelet drugs		120 (30.85%)	35 (24.48%)	0.152
Taken CCB drugs		112 (28.79%)	45 (31.47%)	0.548
Taken ACEI/ARB drugs		117 (30.08%)	48 (33.57%)	0.440
Smoke		170 (43.71%)	69 (48.25%)	0.350
Local anesthesia		326 (83.80%)	110 (76.92%)	0.067
Mean systolic blood pressure (mmHg)		151.860 ± 17.466	153.410 ± 18.916	0.465
Mean diastolic blood pressure (mmHg)		81.630 ± 8.791	81.700 ± 8.546	0.956
anas1 (mm)**		2.966 ± 0.352	3.496 ± 0.327	0.155
A1 (mm)**		3.195 ± 0.935	3.714 ± 0.860	0.724
V1 (mm)**		4.590 ± 1.031	4.895 ± 1.050	0.701
B1 (mm)**		4.983 ± 0.781	5.201 ± 0.684	0.110
F1 (ml/min)**		659.600 ± 337.194	772.936 ± 319.182	0.707
anas3 (mm)***		3.038 ± 0.491	3.374 ± 0.453	0.339
A3 (mm)***		3.500 ± 1.008	3.999 ± 0.944	0.427
V3 (mm)***		5.314 ± 0.997	5.502 ± 1.088	0.428
B3 (mm)***		5.245 ± 0.707	5.472 ± 0.646	0.104
F3 (ml/min)***		874.229 ± 334.380	962.506 ± 305.635	0.130
Red blood cells (10*12/L)		3.488 ± 0.656	3.433 ± 0.622	0.669
Creatinine (umol/L)		730.724 ± 258.812	730.235 ± 249.760	0.672
Urea nitrogen (mmol/L)		20.883 ± 7.534	21.089 ± 7.285	0.841
Blood phosphorus (mmol/L)		1.773 ± 0.606	1.777 ± 0.637	0.270
Albumin (g/L)		40.636 ± 5.273	40.409 ± 5.317	0.525
hemoglobin (g/L)		105.741 ± 19.479	103.735 ± 18.928	0.797
Total cholesterol (mmol/L)		4.025 ± 0.990	3.976 ± 1.024	0.697
total protein (g/L)		71.410 ± 7.910	70.558 ± 7.972	0.423
APTT (s)		30.405 ± 2.917	30.352 ± 2.934	0.878
fibrinogen (g/L)		3.683 ± 0.733	3.678 ± 0.725	0.867
Coronary artery disease*		99 (25.45%)	42 (29.37%)	0.364
Cerebrovascular disease*		64 (16.45%)	25 (17.48%)	0.778
Central Venous Catheterization History*		280 (71.98%)	103 (72.03%)	0.991

### Factors associated with AVF maturation and development of a predictive model

We performed a univariable logistic regression analysis to assess factors associated with AVF maturation (Table 2). The analysis revealed significant differences (*p* < 0.1) in vascular measurements, including mean diastolic blood pressure, anastomotic diameter at 1 month (anas1), arterial diameter at 1 month (A1), venous diameter at 1 month (V1), brachial artery blood flow at 1 month (B1), brachial artery blood flow at 1 month (F1), anastomotic diameter at 3 months (anas3), arterial diameter at 3 months (A3), venous diameter at 3 months (V3), brachial artery diameter at 3 months (B3), and blood flow at 3 months (F3).

**Table 2:** Univariate binary logistic regression in the training cohort.

Parameter	OR	95%CI	*p*
Age(y)	0.996	0.981, 1.013	0.664
Mean systolic blood pressure (mmHg)	1.005	0.993, 1.018	0.383
Mean diastolic blood pressure (mmHg)	1.022	0.997, 1.048	0.079
anas1 (mm)**	0.082	0.038,1.177	<0.001
A1 (mm)**	0.243	0.166, 0.356	<0.001
V1 (mm)**	0.162	0.109, 0.241	<0.001
B1 (mm)**	0.277	0.188, 0.406	<0.001
F1 (ml/min)**	0.991	0.989, 0.993	<0.001
anas3 (mm)***	0.109	0.057,0.209	<0.001
A3 (mm)***	0.259	0.181, 0.372	<0.001
V3 (mm)***	0.150	0.101, 0.222	<0.001
B3 (mm)***	0.226	0.146,0.349	<0.001
F3 (ml/min)***	0.992	0.991,0.994	<0.001
Red blood cells (10*12/L)	1.204	0.865, 1.678	0.271
Creatinine (umol/L)	0.999	0.998, 1.000	0.138
Urea nitrogen (mmol/L)	1.008	0.979, 1.037	0.607
Blood phosphorus (mmol/L)	1.031	0.722, 1.472	0.866
Albumin (g/L)	1.018	0.976,1.061	0.403
hemoglobin (g/L)	1.002	0.991, 1.014	0.685
Total cholesterol (mmol/L)	1.125	0.905, 1.397	0.288
total protein (g/L)	1.010	0.982,1.038	0.484
APTT (s)	1.012	0.940,1.090	0.753
Sex*	0.759	0.491, 1.174	0.215
Diabetes*	1.911	1.229, 2.970	0.004
Hypertension*	1.152	0.628, 2.113	0.648
Coronary artery disease*	0.876	0.530, 1.449	0.607
Cerebrovascular disease*	0.964	0.536, 1.732	0.902
Central Venous Catheterization History*	0.840	0.522, 1.350	0.471
Forearm/upper arm*	1.086	0.571, 2.066	0.802
Dialysistime(y)	1.081	1.003, 1.164	0.040
Left/right*	0.828	0.510, 1.343	0.443
Taken antiplatelet drugs*	1.158	0.729, 1.841	0.535
Taken CCB drugs*	1.002	0.621, 1.614	0.995
Taken ACEI/ARB drugs*	1.222	0.767, 1.945	0.399
Smoke*	1.307	0.846, 2.017	0.228
Anesthesia*	1.106	0.610, 2.006	0.740

*reference group is male; have diabetes; have hypertension; have Coronary Artery Disease; have cerebrovascular disease; have the history of Central venous cathetrization history; Forearm; Left; have the history of taking antiplatelet drugs; have the history of taking CCB drugs; have the history of taking ACEI drugs; smoke; local anrsthsia. **Postoperative ultrasound data were collected at 1 months, Brachial artery diameter (B1), Brachial artery blood flow (F1), Anastomotic diameter (anas1), Internal diameter of anastomotic vein (V1) and Internal diameter of anastomotic artery (A1). ***Postoperative ultrasound data were collected at 3 months, Brachial artery diameter (B3), Brachial artery blood flow (F3), Anastomotic diameter (anas3), Internal diameter of anastomotic vein (V3) and Internal diameter of anastomotic artery (A3).

Significant factors (*p* < 0.1) from the univariable analysis were included in the multivariable logistic regression. The multivariate analysis identified anastomotic diameter at 1 month (anas1), the internal diameter of the anastomotic vein at 1 month (V1), brachial artery blood flow at 1 month (F1), and brachial artery blood flow at 3 months (F3) as independent predictors of AVF maturation (*p* < 0.05) (Table 3).

**Table 3:** Multivariate binary logistic regression in the training cohort.

Parameter	OR	95%CI	*p*
Mean diastolic blood pressure (mmHg)	1.030	0.986, 1.077	0.184
anas1 (mm)**	0.064	0.017, 0.232	<0.001
A1 (mm)**	0.675	0.312, 1.460	0.318
V1 (mm)**	0.529	0.315, 0.889	0.016
B1 (mm)**	1.628	0.726, 3.652	0.237
F1 (ml/min)**	0.996	0.993,0.998	0.001
anas3 (mm)***	1.049	0.383,2.876	0.925
A3 (mm)***	1.749	0.845, 3.621	0.132
V3 (mm)***	0.678	0.370, 1.244	0.209
B3 (mm)***	0.525	0.217, 1.269	0.153
F3 (ml/min)***	0.997	0.994, 0.999	0.005
Diabetes*	1.842	0.886, 3.828	0.102

*reference group is have diabetes. **Postoperative ultrasound data were collected at 1 months, Brachial artery diameter (B1), Brachial artery blood flow (F1), Anastomotic diameter (anas1), Internal diameter of anastomotic vein (V1) and Internal diameter of anastomotic artery (A1). **Postoperative ultrasound data were collected at 1 months, Brachial artery diameter (B1), Brachial artery blood flow (F1), Anastomotic diameter (anas1), Internal diameter of anastomotic vein (V1) and Internal diameter of anastomotic artery (A1). ***Postoperative ultrasound data were collected at 1 months, Brachial artery diameter (B3), Brachial artery blood flow (F3), Anastomotic diameter (anas3), Internal diameter of anastomotic vein (V3) and Internal diameter of anastomotic artery (A3).

### Nomogram construction

**Figure 2:**
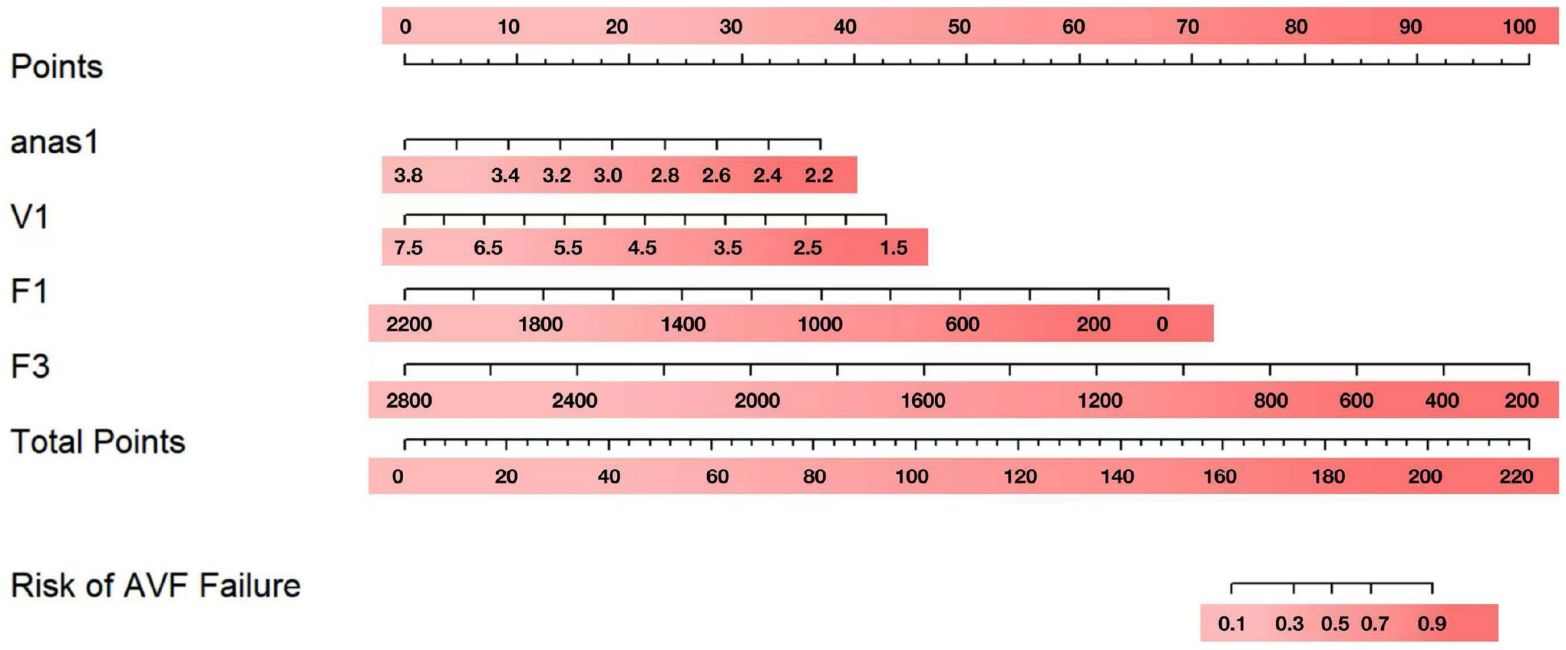
Nomogram for risk prediction of the non-maturation logistic model.

To visualize the above results, nomograms were plotted using anas1, V1, F1, and F3 (Figure 2). After obtaining the four indicators of each patient, a vertical line was drawn on the coordinate axis of each risk indicator to get the corresponding score. These score were then summed to obtain the total score. To obtain the risk percentage of each individual, the total score can be set corresponding to the non-maturation risk on the last coordinate axis.

### Validation of the predictive accuracy of nomogram

**Figure 3:**
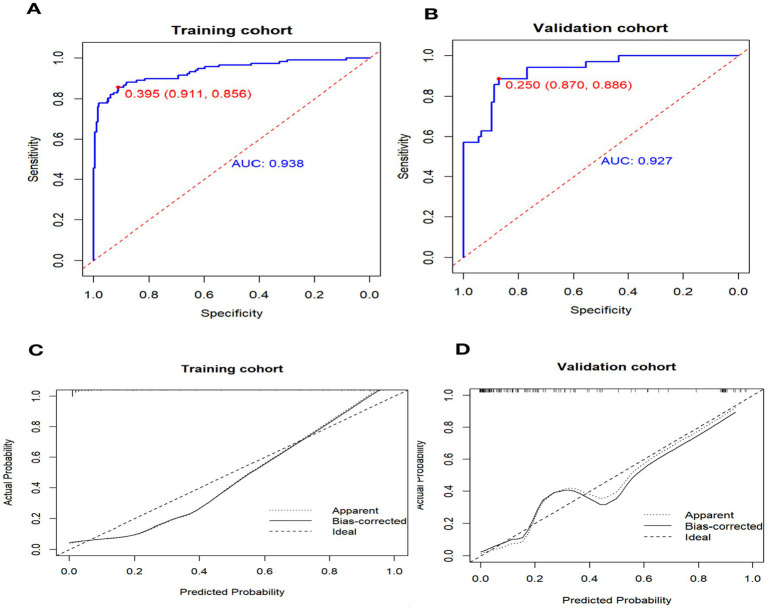
Receiver operating characteristic curve (ROC) and Calibration curve for performance to distinguish individuals with non-maturation (*B* = 1,000 repetitions). AUC, area under the receiver operating characteristic curve.

We used receiver operating characteristic (ROC) analysis to determine the area under the curve (AUC) values for our models. In the training cohort, the nomogram had a significantly high AUC of 0.937 (95% confidence interval, 0.908–0.967), effectively discriminating individuals with non-maturation at 1 month after surgery, with a sensitivity of 0.911 and a specificity of 0.856 (Figure 3A). In the validation cohort, the AUC was 0.927 (95% confidence interval, 0.879–0.975) for patients with non-maturation, with a sensitivity of 0.870 and a specificity of 0.886 (Figure 3B).

The calibration curve illustrates the discrepancy between the real value and the predicted value (Figure 3C). The calibration curve for the validation cohort (Figure 3D) indicates that the predicted value aligns closely with the actual value when the nomogram’s prediction probability exceeds 0.7. However, when the prediction probability of the nomogram falls between 0.4 and 0.6, there is a potential risk of underestimating AVF non-maturation.

## Discussion

In our study, we identified four predictive factors associated with successful AVF maturation: anastomotic diameter at 1 month, internal diameter of anastomotic vein at 1 month, brachial artery blood flow at 1 month, and brachial artery blood flow at 3 months (F3). To the best of our knowledge, insufficient arterial or venous dilation and inadequate vascular remodeling can lead to the failure of an AVF. It should be emphasized that the outward remodeling of the artery plays a crucial role in determining the blood flow in AVFs.

Preexisting arterial intimal hyperplasia may directly affect arterial elastance, potentially limiting blood flow. Concurrently, as the blood flow increases, the diameter of the veins also enlarges, thereby accelerating the maturation process. In our nomogram, we utilized postoperative ultrasound parameters for the first time and confirmed that the artery diameter and the venous diameter at 4 weeks were strongly correlated with AVF maturation. We further developed an effective predictive nomogram, which showed significantly high sensitivity and specificity for distinguishing individuals with and without AVF maturation. This tool might make it easier for clinical staff to assess the risk of AVF maturation better and make informed decisions regarding the appropriate course of action. Currently, Doppler ultrasound is an effective technique in monitoring fistula maturation and is commonly used in clinical practice (17, 18). Access criteria for maturation or intervention in AVF have not yet been clearly defined. The Kidney Disease Outcomes Quality Initiative (KDOQI) Guidelines suggest (14) that fistulas should be evaluated to determine their suitability at 4–6 weeks after creation (15, 19, 20). This raises the question: Can we estimate AVF maturation based on ultrasound parameters at 4 weeks?

The answer seems to be positive. As is well known, a fistula has to be the right size and have sufficient blood flow to enable successful dialysis (21, 22). Indeed, acute revascular remodeling may follow arteriovenous fistula creation (23, 24), as suggested by other studies (25, 26). The majority of the vascular remodeling occurred rapidly, reaching its maximum within 4 weeks after AVF creation, particularly in the diameter of the cephalic vein and the blood flow occurred (8, 27). Additionally, according to certain clinical trials (28), fistula diameters and blood flow rates did not significantly alter in the second, third, or fourth postoperative months. These findings might explain better why we can predict AVF maturation based on ultrasound parameters at 4 weeks. In our study, we compared two contrasting states of maturation. We showed that anastomotic diameter at 1 month, internal diameter of anastomotic vein at 1 month, brachial artery blood flow at 1 month, and brachial artery blood flow at 3 months were significantly different between the two groups (*p* < 0.01). Additionally, the postoperative ultrasound parameters assessed at 4 weeks were found to be highly correlated with AVF maturation. Meanwhile, we established a nomogram model with a predictive efficiency of 0.938, and it was shown that the nomogram model had better congruence and discrimination. This enables us to identify immature AVF early and provide timely interventions, thereby improving outcomes for patients.

The main innovation of this study is as follows: (1) our nomogram included two parameters from internationally accepted standards of the rule of 6 s characteristic for the first time (15, 29), which was different from other prediction models. Lok et al. (28) developed a nomogram that included age over 65 years, female gender, non-white race, and coronary and peripheral arterial disease as the main predictors. Since these factors are distinct from clinical features and are influenced by ethnicity and race, the predictive value for AVF maturation is limited. Twine et al. published a “DISTAL” scoring system for predicting AVF maturation, which combines six predictors: diabetes, ischemic cardiomyopathy, stroke, previous history of AVF surgery, age > 70 years old, and intravenous vessel diameter of <2.0 mm. However, the scoring system mainly targets the internal nasopharyngeal fistula. It has not been further verified in external systems, so it has not been well applied in clinical practice. Bosanquet et al. published the “CAVeA2T2” scoring model to assess the patency of the internal forearm fistula. The model included seven factors, including ipsilateral central vein access, age > 73 years old, anastomosis vein <2.2 mm, previous access history, and intraoperative tremor, for a total of 7 points. AVF with scores >2 was associated with significantly lower patency rates at 6 weeks and 1 year. This score was also not performed in clinical practice due to limitations in statistical methods and not well validated internally. Our predictive models are clearer and more precise in their predictions. (2) Our nomogram enables individualized decision-making for each patient, rather than applying a simple risk stratification to all patients.

As compared to previous studies, which mostly analyzed each parameter separately, using a single predictor is often insufficient, reliable, and scientific. (3) Although the European vascular clinical guidelines considers a diameter of <4 mm and a fistula flow of <500 mL/min to indicate that AVF is unlikely to mature (20, 27, 30). There are still no established criteria for the optimum intervention time among Chinese patients. Our nomogram allows us to quantify and visualize individual patient maturation rates based on fistula diameter and blood flow, helping us determine the best time for intervention. Therefore, our study is unique in that it performed multivariable logistic regression to assess the interaction between the ultrasound measurements and whether to proceed with intervention. This insight may help clinicians make well-informed decisions. In our nomogram, anastomotic length is included as a predictive factor for the maturation of AVF. The major reason for this may be that the larger anastomotic will bring more blood flow and favor adequate dilation of the outflow vein (20, 31). Considering that previous studies on predicting the early maturity of AVF mostly focused on the identification of preoperative relevant clinical features and comorbidities of patients, the results may be more of a prediction of the risk level of AVF surgery itself. Since the imbalance of external and internal vascular remodeling after the establishment of AVF is the main cause of poor maturation, we speculated that postoperative vascular remodeling ability may be a more direct predictor of AVF maturation. Robbin et al.’s study also confirmed that ultrasound variables such as blood flow, diameter, and depth were sufficient for maturation prediction at 6 weeks without considering previous trajectories.

Our study also has several limitations. First, although the data were collected prospectively, the AVF outcomes were analyzed retrospectively, possibly leading to selection bias. Second, as this study reflects the experience of a single center, the findings may not be generalizable to all hemodialysis populations. Third, no analyses of “deep vein,” another important parameter of “6s” for evaluating AVF maturation, were performed in this study. Therefore, further research involving sizable cohorts is needed.

In summary, our nomogram, which integrates anastomotic diameter at 1 month, internal diameter of the anastomotic vein at 1 month, brachial artery blood flow at 1 month, and brachial artery blood flow at 3 months, provides valuable insights for accurately and individually assessing the risk of AVF maturation failure. This tool can help clinicians in implementing timely and targeted intervention measures. However, these preliminary findings require validation through future multi-center prospective studies due to the limitations of the current prediction model.

## Data Availability

The original contributions presented in the study are included in the article/supplementary material, further inquiries can be directed to the corresponding author.
